# Vocal deviation in individuals with suggestive signs and symptoms of laryngopharyngeal reflux

**DOI:** 10.1590/2317-1782/20212019065

**Published:** 2022-02-25

**Authors:** Ana Julia Sartori, Régis Dewes, Glaucya Madazio, Felipe Moreti, Mara Behlau

**Affiliations:** 1 Centro de Estudos da Voz – CEV - São Paulo (SP), Brasil.; 2 RD Serviços Médicos - Lajeado (RS), Brasil.

**Keywords:** Voice, Dysphonia, Laryngopharyngeal Reflux, Signs and Symptoms, Speech, Language and Hearing Sciences

## Abstract

**Purpose:**

Verify and compare vocal deviation in quality, vocal symptoms and reflux symptom index in patients with clinical diagnosis of laryngopharyngeal reflux (LPR).

**Methods:**

100 individuals of both genders participated in this prospective study, aged between 18 and 60 years old, who presented signs of LPR in the nasofibrolaryngological exam. Participants answered the Reflux Symptom Index (RSI) questionnaire to determine the reflux index and the Voice Symptom Scale (VoiSS). Their voices were recorded for the auditory-perceptual assessment. Three speech therapists with voice experience were contacted and the most reliable one was maintained.

**Results:**

100 examined voices, 34 were classified as adapted and 66 as deviated. The predominant vocal quality type was rough and a slight degree of deviation. The average score on VoiSS and RSI of individuals with deviated voice is significantly higher than the adapted voice group on both protocols (p<0.01). The symptom reported with most frequency and intensity, in both analyses, was throat clearing. There were statistically significant differences once analyzed the vocal quality types by pairs: rough-adapted (p=0.0021) and tense-adapted (p=0.0075) on VoiSS, and rough-adapted (p=0.001) on RSI.

**Conclusion:**

Individuals with deviated voice reported higher occurrence of LPR related vocal signals and symptoms measured by VoiSS and RSI. The numerous theories about the disease do not make possible a single conclusion on the subject. Further studies are needed in the area to assist the professional in the diagnosis and treatment of the RLF patient.

## INTRODUCTION

The voice identifies humans not only by sex, age, and physical type, but also reveals their emotional state and personality characteristics. For it to be produced, a complex and interdependent action of muscles and the integrity of the vocal tract are needed^(1)^. Many causes alter the vocal quality, including some diseases such as laryngopharyngeal reflux (LPR), considered one of the most common extraesophageal manifestations of gastroesophageal reflux disease, which affects the larynx and pharynx^(2)^.

LPR represents a displacement of the stomach contents to the larynx and pharynx^(3,4)^, being considered a relatively common disease that affects 50% of the population and is pointed out as one of the most important causal factors for the onset of dysphonia^(5)^.

The laryngeal tissues are protected from reflux damage due to the pH regulation of type III carbonic anhydrase enzyme, which catalyzes carbon dioxide to produce bicarbonate, protecting the posterior laryngeal tissues from acid reflux. This enzyme is expressed at high levels in a normal larynx; however, it is absent in 64% of laryngeal tissue biopsy samples in LPR patients^(6,7)^. In the esophagus, 50 episodes of reflux per day are considered normal, whereas, in the larynx, three episodes can already cause damage^(8)^.

Due to the effects caused by acid reflux, such as coughing, throat clearing, and even acid itself, the vocal folds can change their constitution and thus appear typical lesions^(9)^. Due to the change in the vibratory process of the vocal folds, the aerodynamic values (maximum phonation time) and acoustic (jitter and shimmer) appear altered in patients with LPR compared to healthy individuals^(10)^, indicating disturbance and decreased laryngeal control during phonation^(11)^. The signs observed in patients with LPR are diffuse laryngeal edema, hyperemia, interarytenoid hypertrophy, contact ulcer, granuloma or granulation, and thickening of the posterior region of the glottis.

Regarding the symptoms, the presence of pharyngeal globe, retrosternal burning, chronic throat clearing, posterior rhinorrhea, halitosis, hoarseness, vocal fatigue, vocal breaks, dysphagia, regurgitation, chronic cough, wheezing, respiratory obstruction, and paroxysmal laryngospasm are reported. Dysphonia is the most present clinical symptom and is characterized by muscle tension, sudden vocal attack, use of basal voice, limited modulation, and hoarseness; followed by pharyngeal globe, throat clearing, and coughing^(12,13)^. However, these symptoms can also be the result of other etiological factors such as smoking, allergies, sinusitis, and inhaled medications, which makes the etiology of these occurrences generally non-specific, with a wide spectrum of differential diagnoses^(2)^.

LPR is a multifactorial disease that generates changes in both organic matter and vocal function, requiring a multidisciplinary assessment. Patients’ treatment is done through specific medications and lifestyle changes^(14,15)^.

When the professional evaluates the patient with complaints and laryngological changes without evolution with the treatment performed, he must consider the diagnostic suspicion of LPR.

This study aimed to verify and correlate the deviation of vocal quality, vocal symptoms, and reflux symptoms index in patients with clinical diagnoses suggestive of LPR. Given the numerous theories about LPR and the difficulty in diagnosing it clearly and objectively, it is intended to contribute with information that led professionals to identify and analyze clinical findings as auxiliary tools in patient diagnosis.

## METHODS

This research was approved by the Ethics Committee under opinion No. 1.545.346 and all participants signed the Informed Consent Form.

One hundred individuals were evaluated with a mean age of 43.4 years (SD=12.8), a median of 45.5 and ranging from 19 to 60 years old, 63 women and 37 men, who reported complaints related to reflux (hoarseness, change in voice, throat clearing, secretions in the throat, difficulty in swallowing and/or breathing, coughing, feeling of something stuck in the throat and/or heartburn) in the consultation with the otolaryngologist. They were submitted to nasofibrolaryngoscopy exam and, when signs suggestive of reflux were observed, they were invited to participate in the research.

Inclusion criteria were individuals of both sexes, between 18 and 60 years old and who presented a medical diagnosis suggestive of reflux. Patients undergoing drug treatment for reflux, with a medical diagnosis of active psychiatric and/or psychological, respiratory, and metabolic diseases, or with laryngeal lesion due to speech trauma and who underwent head and neck surgery, were excluded from the study.

After nasofibrolaryngoscopy, all participants completed two protocols: the Reflux Symptom Index (RSI)^(16)^ and Voice Symptom Scale (VoiSS)^(17,18)^. The questionnaires were applied and explained by the researcher and the patients were helped to fill them out, when necessary. Then, they were submitted to voice recording.

For nasofibrolaryngoscopy, performed by the same otorhinolaryngologist, 3 sprays of anesthetic spray Lidocaine, a 2% solution, were applied in each nostril, minutes before the exam. The videoendoscope used was a 3.7mm VNL 1170K model from the Pentax brand. As diagnostic criteria suggestive of reflux, the physician observed the presence of one or more characteristic signs: vocal fold edema, laryngeal ventricle obliteration, pseudo sulcus, hyperemia, endolaryngeal secretion, and granulation tissue.

The RSI^(16)^consists of nine questions that investigate the presence of LPR symptoms. The individuals were asked to answer whether or not they presented, in the last month, some of the investigated items: hoarseness or voice problems, throat clearing, excessive secretion in the throat or nose, difficulty in swallowing food, liquids, or pills, coughing after eating or after lying down, breathing difficulties or choking episodes, excessive coughing, feeling of something stuck in the throat and heartburn, chest pain, indigestion or stomach acid in the mouth. If so, the intensity is scored from 0 (zero) to 5 (five). The maximum score is 45 points and is considered altered when the total value is equal to or greater than 13 points and normal when less than this.

The VoiSS^(17,18)^ assesses the self-perception of vocal symptoms and the impact of the voice problem. Individuals were instructed to answer 30 questions, divided into three domains: Impairment (fifteen questions), Emotional (eight), and Physical (seven). Each question is scored from zero to four, according to the frequency of occurrence: never, rarely, sometimes, almost always, and always, with scores calculated by the simple sum of the points. The higher the scores in this protocol, the greater the perception of the general level of voice alteration about the impairment in voice use, emotional reactions, and physical symptoms. In the total score, the cutoff score is 16 points^(18,19)^, while in the Impairment, Emotional and Physical domains the values are 11.5, 1.5, and 6.5, respectively. In this study, the total score, the specific ones, and the three questions with the greatest deviation were evaluated.

The auditory-perceptual assessment classified the voices according to the type of predominance of vocal quality (adapted, rough, breathy, or tense) and the degree of change in the predominant quality through the Visual Analog Scale (VAS)^(20)^ and its corresponding numerical. Initially, 3 speech-language therapists with experience in the voice area were contacted and the one with the greatest reliability was maintained. For this, 20% of the samples were repeated, at random, to verify the intra-rater reliability (0.975). Voices were evaluated in a silent environment, using headphones made by Sony, model MDR-ZX110, in an Acer computer.

For the auditory-perceptual assessment, the speech material was obtained by recording the emission of the vowel /Ɛ/ sustained and counting numbers from 1 to 10. In this study, we chose to use connected speech and sustained vowels. The chained speech was used to observe the presence of resonant and phonoarticulatory adjustments, the elements of pneumophonic coordination, and prosody. The option to choose both tasks, sustained vowel and connected speech, was based on exploring not only the deviation of vocal quality. Both tasks, taken together, help to better judge the degree of vocal quality deviation within the context of the use of voice in oral communication. The voices were recorded in a silent but not acoustically treated environment. A computer from the Acer brand was used, with an Auricular microphone Karsect HT-9 and an Andrea PureAudio USB adapter. The program used for recording was FonoView, by CTS Informática.

We used the VAS to determine the degree of change in the predominant quality^(20)^– 100mm line, where the left end corresponds to the absence of vocal alteration and the right end the maximum degree - and its numerical counterpart, the Numerical Scale (NS)^(20)^, which is divided into four tracks according to vocal deviation: 0 for normal voice quality variability, 1 for mild to moderate deviation, 2 for moderate deviation, and 3 for severe deviation.

The calculations and descriptive analysis of the results were generated using the SPSS software version 23. We used chi-square to verify the associations between type of vocal quality and gender, and a t-test to compare possible differences between the type of vocal quality and age. The Shapiro-Wilk test was used to study the normality of VoiSS and RSI values. To compare the VoiSS and RSI results between individuals with adapted voices and with deviated voices, as well as between individuals with different types of predominant voices, the Wilcoxon test was used - a non-parametric test used to compare two related samples - with the level of 5% significance.

## RESULTS

Regarding the 100 voices evaluated, 63 were female and 37 were male. Of the total, 34 (34%) were classified as adapted, being 23 women and 11 men; and 66 (66%) deviated, totaling 40 women and 26 men with an average age of 44.8 years. Of the deviated ones, 53 (80.3%) had a predominantly rough quality. Regarding the degree, 42 subjects were identified with mild vocal deviation (grade 1 in VAS), totaling 63.6% of deviated voices.

The most prevalent laryngeal findings found in the nasofibrolaryngological exam were vocal fold edema, observed in 98 (98%) of the patients, and hyperemia, observed in 95 (95%) of the patients evaluated. On the other hand, only 15 subjects (15%) had granulation tissue.

To verify the association of gender in adapted and deviated voices, we performed the chi-square test of association and observed no statistical evidence (p=0.521). To compare the mean age of individuals with adapted and deviated voices, we used the t-test, adopting a significance level of 5%, and found no statistical evidence (p=0.11). (Table 1).

**Table 1:** Mean age of individuals with adapted and deviated voices

**Voice**	**N**	**Average**	**Standard Deviation**	**P-value**
**Adapted**	34	40.56	11.64	
				0.11
**Deviated**	66	44.89	13.26	

t-test for independent samples between age and vocal characteristics

We used descriptive data analysis to evaluate the results obtained in the VoiSS and RSI protocols. In the VoiSS, 70 subjects had altered total score (>16 points), of which 51 had altered voices, 40 of which were rough, 6 breathy, and 4 tense. The others presented an adapted voice. Among individuals with normal scores on the VoiSS, 15 had adapted voice and 15 deviated, 12 of which were of the rough type. The three questions with the greatest deviation were: “Do you cough or clean your throat?”, “Does it feel as if there is something stuck in your throat?” and “Do you have a lot of phlegm in your throat?”. The physical domain showed the greatest deviation.

In the RSI, 61 individuals had an altered score (≥ 13 points) with vocal alteration with a predominance of the rough type in 39 subjects, 4 breathy and 4 tenses. The others presented an adapted voice. Among individuals with normal scores on the RSI, 20 had adapted voice and 19 deviated, 13 of which were of the rough type.

When comparing the mean total scores, both VoiSS and RSI, in adapted and deviated voices, it was observed that the mean of individuals with deviated voices is significantly higher than in the group with adapted voices in both protocols (p<0.01). (Table 2).

**Table 2:** Total VoiSS and RSI scores of individuals with adapted and deviated voices

**Voice**	**N**	**Average**	**Mean**	**Standard Deviation**	**P-value**
**VoiSS**					
**Adapted**	34	19.62	16.5	12.15	
					<0.01
**Deviated**	66	31.11	30.0	16.47	
**RSI**					
**Adapted**	34	11.29	11	5.27	
					<0.01
**Deviated**	66	17.04	16	6.99	

Wilcoxon test for independent samples for VoiSS and RSI

**Caption:** VoiSS = Voice Symptom Scale; RSI = Reflux Symptom Index

Descriptive statistics of the total VoiSS and RSI values were analyzed for the predominant types of vocal quality and, to test whether there was any difference in the values, a non-parametric comparison was used for each pair. We observed statistically significant differences when analyzing the pairs: rough-adapted (p=0.0021) and tense-adapted (p=0.0075) in the VoiSS, and rough-adapted (p=0.001) in the RSI (Table 3); noting a direct relationship between the increased scores in the VoiSS and RSI with the type of vocal quality.

**Table 3:** Non-parametric comparison for each voice quality parameter pair in VoiSS and RSI values

	**Adapted**	**Rough**	**Breathy**
**VoiSS**			
**Adapted**	-		
**Rough**	0.0021*	-	
**Breathy**	0.0799	0.7999	-
**Tense**	0.0075*	0.2588	0.5192
**RSI**			
**Adapted**	-		
**Rough**	<0.001*	-	
**Breathy**	0.4440	0.5197	-
**Tense**	0.0948	0.8407	0.1039

Wilcoxon Method

* Statistically significant differences, with alpha at 0.05

**Caption:** VoiSS = Voice Symptom Scale; RSI = Reflux Symptom Index

## DISCUSSION

The present study verified the deviation of vocal quality, vocal symptoms, and reflux symptoms index in patients with clinical diagnosis suggestive of LPR attended at an otorhinolaryngology clinic in the city of Lajeado - RS and, based on the results obtained, statistically analyzed the data and the possible correlations between them.

Many studies have been conducted since the first publication on LPR, but controversies regarding the pathology remain. A recent study considers the definition of LPR reported so far to be incomplete, as it believes that the alteration caused does not only include the laryngopharyngeal mucosa but the entire mucosa of the aerodigestive tract. The multifactorial origin of symptoms is also considered to be the result of neuroreflexive signaling and compensatory vagal responses^(21)^.

The relationship between reflux and voice has been evolving over the last 40 years and it is increasingly common to attribute vocal alterations to this pathology, especially in the absence of other obvious etiologies. So far what is known is that there is a relationship between them^(22)^. A study^(23)^ carried out with 121 individuals showed a record of an altered general degree, breathiness, tension, and instability in the group with the presence of LPR symptoms. In addition to these findings, the present study also found a greater number of patients with deviated voices, with a predominantly rough voice and a mild degree of alteration.

The study observed a higher occurrence of female patients, as reported in the literature that observed that women have more reflux complaints than men^(24)^. The mean age of women with deviated voices was 41.1 years and 47.2 years for men, which agrees with the study that found that older individuals are more likely to present LPR findings compared to younger subjects^(25)^.

LPR affects laryngeal behavior and as a consequence of chemical irritation of the refluxed material, clinical signs such as mucosal edema and hyperemia are found in laryngological exams^(26)^. In agreement with the literature, we observed that 98% of the patients in this study had vocal fold edema and 95% hyperemia.

Hoarseness is one of the main symptoms among the main complaints reported by patients with LPR, being present in up to 50% of subjects with vocal disorders. In addition to this, they also report mucus and chronic cough^(5)^. In another study with 39 individuals with LPR signs, more than 70% reported symptoms such as throat clearing and dysphonia^(27)^. Analyzing each question of the protocols applied in this study (VoiSS and RSI) individually, it was observed that the items related to throat clearing were mentioned in greater intensity and frequency by patients, in both assessments. Mucus can be caused by edema in the retrocricoid region and generate an increase in local inflammation, compromising the phonatory function and may form ulcers or granulations due to contact in the region of the vocal processes^(9)^.

In VoiSS, the highest mean (2.25) refers to question 7, “Do you cough or clean your throat?”, followed by 19 (2.14), “Do you have a lot of phlegm in your throat?” and from question 11 (1.75) “Does it feel as if there is something stuck in your throat?”. Of the ten questions that most influenced the worsening of vocal symptoms, six were from the physical dimension, that is, the questions referring directly to the voice (impairment dimension) did not intervene in the increase in the total VoiSS score, justifying the findings that some patients identified with adapted voices presented altered results in this protocol and the complexity when dealing with this subject. In the RSI^(16)^, the highest mean (3.20) refers to the item “Clearing your throat” – question 2, followed by question 3 “Excess throat mucus or postnasal drip” (2.87). These results are in line with the findings found in the study^(28)^ where the same protocol for evaluation was used and it was found that the same questions corresponded to 85.7% and 82.9% of symptoms, respectively. The high number of subjects found with an altered score was expected since the presence of signs and symptoms suggestive of LPR was used as a criterion for inclusion in the study.

The association between signs suggestive of LPR and the presence of vocal alterations is still controversial. As in this research, the studies point to a relationship, but do not prove the causal relationship between them^(5,29,30)^. The vast difference that exists between studies on LPR, concerning epidemiology, clinical signs, diagnosis, and treatment, makes it difficult to establish a single conclusion on the subject. When using the laryngological exam instead of pH-metry to identify signs suggestive of reflux, we may have created a limitation in our study, since the latter is considered the gold standard in the diagnosis of reflux. However, the study makes an important contribution to the area of voice and laryngopharyngeal reflux due to its representative number of subjects and the form of multidimensional vocal assessment, with information from laryngeal assessment, self-assessment protocols, and preceptive-auditory voice assessment, bringing robustness to their results.

## CONCLUSION

Most subjects with a diagnosis suggestive of LPR had clinically measured vocal deviations. Individuals with deviated voices reported a higher occurrence of vocal symptoms, vocal handicap, and unpleasant sensations related to the LPR measured by the VoiSS and RSI.
