# Supplementary material for: Vocal deviation in individuals with suggestive signs and symptoms of laryngopharyngeal reflux
Source: Codas. 2022 Feb 25;34(4):e20190065. doi: 10.1590/2317-1782/20212019065 (PMC9886118; doi:10.1590/2317-1782/20212019065)
Supplement: Translation

# Translation: Desvio vocal em indivíduos com sinais e sintomas sugestivos de refluxo laringofaríngeo

**Authors:** Ana Julia Sartori, Régis Dewes, Glaucya Madazio, Felipe Moreti, Mara Behlau

**Affiliations:** 1 Centro de Estudos da Voz – CEV - São Paulo (SP), Brasil.; 2 RD Serviços Médicos - Lajeado (RS), Brasil.

**Keywords:** Voz, Disfonia, Refluxo Laringofaríngeo, Sinais e Sintomas, Fonoaudiologia

## Abstract

**Objetivo:**

verificar e comparar o desvio da qualidade vocal, sintomas vocais e índice de sintomas de refluxo em pacientes com diagnóstico clínico sugestivo de refluxo laringofaríngeo (RLF).

**Método:**

participaram deste estudo prospectivo 100 indivíduos de ambos os sexos, com faixa etária entre 18 e 60 anos que apresentaram sinais de RLF no exame nasofibrolaringológico. Os participantes responderam ao questionário Índice de Sintomas do Refluxo Faringo-Laríngeo (ISRFL) para determinar presença de sintomas de refluxo e a Escala de Sintomas Vocais (ESV). Tiveram suas vozes gravadas para a avaliação perceptivoauditiva. Foram contatadas três fonoaudiólogas com experiência em voz e manteve-se a de maior confiabilidade.

**Resultados:**

100 vozes avaliadas, 34 eram adaptadas e 66 desviadas. O tipo de qualidade vocal predominante foi rugoso e o grau de desvio leve. A média dos escores no ESV e ISRFL dos indivíduos com vozes desviadas foi significativamente maior que no grupo de vozes adaptadas em ambos os protocolos (p<0,01). O sintoma relatado com maior frequência e intensidade, em ambas as avaliações, foi pigarro. Houve diferenças estatisticamente significativas quando analisados os pares dos tipos de qualidade vocal: rugosa-adaptada (p=0,0021) e tensa-adaptada (p=0,0075) no ESV, e rugosa-adaptada (p=0,001) no ISRFL.

**Conclusão:**

indivíduos com vozes desviadas referiram maior ocorrência de sinais e sintomas vocais relacionadas ao RLF mensurados pela ESV e ISRFL. As inúmeras teorias a respeito da doença não tornam possível uma única conclusão sobre o assunto. São necessários novos estudos na área a fim de auxiliar o profissional no diagnóstico e tratamento do paciente com RLF.

## INTRODUÇÃO

A voz identifica o homem não somente quanto ao sexo, idade e tipo físico, mas também revela seu estado emocional e características de personalidade. Para que seja produzida, é necessária uma complexa e interdependente ação de músculos e a integridade do aparelho fonador^(1)^. Muitas são as causas que alteram a qualidade vocal, entre elas, algumas doenças como o refluxo laringofaríngeo (RLF), considerado uma das manifestações extraesofágicas mais comuns da doença do refluxo gastroesofágico, o qual acomete a laringe e faringe^(2)^.

O RLF representa um deslocamento do conteúdo do estômago à laringe e faringe^(3,4)^, sendo considerado uma doença relativamente comum que afeta 50% da população e é apontado como um dos fatores causais mais importantes para o surgimento de uma disfonia^(5)^.

Os tecidos da laringe são protegidos de danos causados pelo refluxo devido à regulação do pH da enzima anidrase carbônica do tipo III, a qual catalisa o dióxido de carbono para a produção de bicarbonato, protegendo os tecidos da porção posterior laríngea do refluxo ácido**.** Esta enzima é expressa em níveis elevados em uma laringe normal, entretanto, encontra-se ausente em 64% das amostras de biópsias de tecidos da laringe em pacientes com RLF^(6,7)^. No esôfago, 50 episódios de refluxo por dia são considerados normais, ao passo que na laringe três episódios já podem causar danos^(8)^.

Devido aos efeitos provocados pelo refluxo ácido, como a tosse, o pigarro e até mesmo o próprio ácido, as pregas vocais podem sofrer alterações na sua constituição e assim surgirem lesões típicas^(9)^. Em função da mudança no processo vibratório das pregas vocais os valores aerodinâmicos (tempo máximo de fonação) e acústicos (*jitter* e *shimmer*) aparecem alterados em pacientes com RLF comparados a indivíduos saudáveis^(10)^, indicando perturbação e diminuição do controle laríngeo durante a fonação^(11)^. Os sinais observados em pacientes com RLF são edema laríngeo difuso, hiperemia, hipertrofia interaritenóidea, úlcera de contato, granuloma ou granulação e espessamento da região posterior da glote.

Quanto aos sintomas, são relatados presença de globo faríngeo, queimação retroesternal, pigarro crônico, rinorreia posterior, halitose, rouquidão, fadiga vocal, quebras vocais, disfagia, regurgitação, tosse crônica, chiado no peito, obstrução respiratória e laringoespasmo paroxístico, sendo a disfonia o sintoma clínico mais presente e caracterizada por tensão muscular, ataque vocal brusco, uso de voz basal, modulação limitada e rouquidão; seguida pelo globo faríngeo, pigarro e tosse^(12,13)^. Entretanto, estes sintomas também podem ser resultantes de outros fatores etiológicos como tabagismo, alergias, sinusites e medicamentos inalados, o que torna a etiologia destas ocorrências geralmente não específica, com um amplo espectro de diagnósticos diferenciais^(2)^.

O RLF é uma doença multifatorial que gera mudanças tanto na questão orgânica quanto na função vocal, sendo necessária a avaliação multidisciplinar. O tratamento aos pacientes se faz por meio de medicamentos específicos e mudanças no estilo de vida^(14,15)^.

É necessário que o profissional, ao avaliar o paciente com queixas e alterações laringológicas sem evolução com o tratamento realizado, considere a suspeita diagnóstica de RLF.

O objetivo deste estudo foi verificar e correlacionar o desvio da qualidade vocal, sintomas vocais e índice de sintomas de refluxo em pacientes com diagnóstico clínico sugestivo de RLF. Diante das inúmeras teorias a respeito do RLF e da dificuldade em diagnosticá-lo de forma clara e objetiva, pretende-se contribuir com informações que levem o profissional a identificar e analisar os achados clínicos como ferramentas auxiliares no diagnóstico do paciente.

## MÉTODO

Esta pesquisa foi aprovada pelo Comitê de Ética sob parecer nº 1.545.346 e todos os participantes assinaram o Termo de Consentimento Livre e Esclarecido.

Foram avaliados 100 indivíduos com média de idade de 43,4 anos (DP=12,8), mediana de 45,5 e variando de 19 a 60 anos, sendo 63 mulheres e 37 homens, que relataram queixas referentes ao refluxo (rouquidão, alteração na voz, pigarro, secreção na garganta, dificuldade de deglutição e/ou respiratória, tosse, sensação de algo parado na garganta e/ou azia) na consulta com o médico otorrinolaringologista. Estes foram submetidos ao exame de nasofibrolaringoscopia e, quando observados sinais sugestivos de refluxo, eram convidados a participar da pesquisa.

Como critérios de inclusão, foram inseridos indivíduos de ambos os sexos, com faixa etária entre 18 e 60 anos e que apresentaram diagnóstico médico sugestivo de refluxo. Pacientes em tratamento medicamentoso para refluxo, com diagnóstico médico de doenças psiquiátricas e/ou psicológicas, respiratórias e metabólicas ativas, ou ainda com lesão laríngea por fonotrauma e que realizaram cirurgia de cabeça e pescoço, foram excluídos da pesquisa.

Após a nasofibrolaringoscopia, todos os participantes preencheram dois protocolos: Índice de Sintomas do Refluxo Faringo-Laríngeo (ISRFL)^(16)^ e Escala de Sintomas Vocais (ESV)^(17,18)^. Os questionários foram entregues e explicados pela pesquisadora e os pacientes receberam auxílio no preenchimento, quando necessário. Em seguida, foram submetidos à gravação da voz.

Para a realização da nasofibrolaringoscopia, executada pelo mesmo médico otorrinolaringologista, foram aplicadas 3 borrifadas de *spray* anestésico Lidocaína solução 2%, em cada narina, minutos antes da realização do exame. O videoendoscópio utilizado foi do modelo VNL 1170K de 3.7mm da marca *Pentax*. Como critério de diagnóstico sugestivo de refluxo, o médico observou a presença de um ou mais sinais característicos: edema de prega vocal, obliteração de ventrículo laríngeo, pseudosulco, hiperemia, secreção endolaríngea e tecido de granulação.

O protocolo ISRFL^(16)^ é composto por nove questões que investigam a presença de sintomas do RLF. Os indivíduos foram orientados a responder se apresentaram ou não, no último mês, alguns dos itens investigados: rouquidão ou problemas de voz, pigarro, secreção excessiva na garganta ou no nariz, dificuldade para engolir comida, líquidos ou comprimidos, tosse após ter comido ou depois de deitar-se, dificuldades respiratórias ou episódios de engasgos, tosse excessiva, sensação de alguma coisa parada na garganta e azia, dor no peito, indigestão ou ácido do estômago na boca. Em caso positivo, a intensidade é graduada de 0 (zero) a 5 (cinco). O escore máximo é de 45 pontos e é considerado alterado quando o valor total for igual ou superior a 13 pontos e normal quando inferior a este.

O protocolo ESV^(17,18)^avalia a autopercepção de sintomas vocais e o impacto do problema de voz. Os indivíduos foram instruídos a responder 30 questões, divididas em três domínios: Limitação (quinze questões), Emocional (oito) e Físico (sete). Cada pergunta é pontuada de zero a quatro, de acordo com a frequência de ocorrência: nunca, raramente, às vezes, quase sempre e sempre, com escores calculados pela soma simples dos pontos. Quanto maiores os escores neste protocolo, maior é a percepção do nível geral de alteração de voz no que diz respeito à limitação no uso da voz, reações emocionais e sintomas físicos. No escore total, a nota de corte é de 16 pontos^(18,19)^, enquanto nos domínios Limitação, Emocional e Físico os valores são 11,5, 1,5 e 6,5, respectivamente**.** Neste estudo foi avaliado o escore total, os específicos e as três questões com maior desvio.

A avaliação perceptivoauditiva classificou as vozes quanto ao tipo de predominância da qualidade vocal (adaptada, rugosa, soprosa ou tensa) e ao grau de alteração da qualidade predominante por meio da Escala Analógica Visual (EAV)^(20)^e sua correspondente numérica. Inicialmente, foram contatadas 3 fonoaudiólogas com experiência na área de voz e manteve-se a de maior confiabilidade. Para isso, 20% das amostras foram repetidas, de forma aleatória, a fim de verificar a confiabilidade intra-avaliador (0,975). As vozes foram avaliadas em ambiente silencioso, por meio de fones de ouvido da marca Sony, modelo MDR-ZX110, em um computador da marca *Acer.*

Para a avaliação perceptivoauditiva o material de fala foi obtido através da gravação da emissão da vogal /Ɛ/ sustentada e a contagem de números de 1 a 10. Neste estudo, optou-se por utilizar a fala encadeada e a vogal sustentada. Utilizou-se a fala encadeada para observar a presença de ajustes ressonantais e fonoarticulatórios, os elementos de coordenação pneumofônica e a prosódia. A opção de escolha de ambas as tarefas, vogal sustentada e fala encadeada, baseou-se na exploração não somente do desvio da qualidade vocal. Ambas as tarefas, em conjunto, auxiliam o melhor julgamento do grau de desvio da qualidade vocal dentro do contexto do uso da voz na comunicação oral. A gravação das vozes foi realizada em ambiente silencioso, porém não acusticamente tratado. Utilizou-se um computador da marca *Acer*, com microfone Auricular Karsect HT-9 e adaptador Andrea PureAudio USB. O programa utilizado para a gravação foi o FonoView, da CTS Informática.

Para determinar o grau de alteração da qualidade predominante, utilizou-se a EAV^(20)^– linha de 100 milímetros, onde a extremidade esquerda corresponde a ausência de alteração vocal e a direita o grau máximo - e sua correspondente numérica, a Escala Numérica (EN)^(20)^, que se divide em quatro faixas de acordo com o desvio vocal: 0 para variabilidade normal da qualidade de voz, 1 para desvio leve a moderado, 2 para desvio moderado e 3 para desvio intenso.

Os cálculos e análise descritiva dos resultados foram gerados com o auxílio do software SPSS versão 23. Para verificar as associações entre tipo de qualidade vocal e gênero foi utilizado o teste qui quadrado de associação e, para comparar as possíveis diferenças entre tipo de qualidade vocal e idade, foi utilizado o teste t. Utilizou-se o teste Shapiro-Wilk para o estudo de normalidade dos valores do ESV e ISRFL. Para comparar os resultados do ESV e ISRFL entre indivíduos com voz adaptada e com voz desviada, assim como entre os indivíduos com diferentes tipos de vozes predominantes, foi utilizado o teste de Wilcoxon – teste não paramétrico utilizado para comparar duas amostras relacionadas - com nível de significância de 5%.

## RESULTADOS

Referente as 100 vozes avaliadas, 63 eram femininas e 37 masculinas. Do total, 34 (34%) foram classificadas como adaptadas, sendo 23 de mulheres e 11 de homens; e 66 (66%) desviadas, totalizando 40 mulheres e 26 homens com faixa etária média de 44,8 anos. Das desviadas, 53 (80,3%) apresentaram qualidade predominantemente rugosa. Com relação ao grau, 42 sujeitos foram identificados com desvio vocal leve (grau 1 na EAV), totalizando 63,6% das vozes desviadas.

Os achados laríngeos de maior prevalência encontrados no exame nasofibrolaringológico foi o edema de prega vocal, observado em 98 (98%) dos pacientes e a hiperemia, observada em 95 (95%) dos pacientes avaliados. Em contrapartida, apenas 15 sujeitos (15%) apresentaram tecido de granulação.

Para verificar a associação de gênero nas vozes adaptadas e desviadas, foi realizado o teste qui quadrado de associação, onde não foram observadas evidências estatísticas (p=0,521). Para comparar a média de idade dos indivíduos com vozes adaptadas e desviadas, foi utilizado o teste t, adotando-se um nível de significância de 5%, onde não foram constatadas evidências estatísticas (p=0,11). (Tabela 1).

**Tabela 1:** Médias de idade dos indivíduos com vozes adaptadas e desviadas

**Voz**	**N**	**Média**	**Desvio Padrão**	**Valor de p**
**Adaptada**	34	40,56	11,64	
				0,11
**Desviada**	66	44,89	13,26	

Teste-t para amostras independentes entre idade e características vocais

Para avaliar os resultados obtidos nos protocolos ESV e ISRFL foi utilizada a análise descritiva dos dados. No ESV, 70 sujeitos apresentaram escore total alterado (>16 pontos), destes 51 tinham vozes alteradas, sendo 40 do tipo rugosa, 6 soprosa e 4 tensa. Os demais apresentaram voz adaptada. Entre os indivíduos com escore normal no ESV, 15 apresentaram voz adaptada e 15 desviadas, sendo 12 do tipo rugosa. As três questões com maior desvio foram: “você tosse ou pigarreia?”, “você sente alguma coisa parada na garganta?” e “você tem muita secreção ou pigarro na garganta?”. O domínio Físico apresentou maior desvio.

No ISRFL, 61 indivíduos apresentaram escore alterado (≥ 13 pontos) com alteração vocal com predominância do tipo rugosa em 39 sujeitos, 4 soprosa e 4 tensa. Os demais apresentaram voz adaptada. Entre os sujeitos com escore normal no ISRFL, 20 apresentaram voz adaptada e 19 voz desviada, sendo 13 do tipo rugosa.

Ao comparar as médias dos escores totais, tanto do ESV como do ISRFL, nas vozes adaptadas e desviadas, observou-se que a média dos indivíduos com vozes desviadas é significativamente maior que no grupo de vozes adaptadas em ambos os protocolos (p<0,01). (Tabela 2).

**Tabela 2:** Escores totais da ESV e ISRFL dos indivíduos com vozes adaptadas e desviadas

**Voz**	**N**	**Média**	**Mediana**	**Desvio Padrão**	**Valor de p**
**ESV**					
**Adaptada**	34	19,62	16,5	12,15	
					<0,01
**Desviada**	66	31,11	30,0	16,47	
**ISRFL**					
**Adaptada**	34	11,29	11	5,27	
					<0,01
**Desviada**	66	17,04	16	6,99	

Teste de Wincoxon para amostras independentes para o ESV e ISRFL

**Legenda:** ESV = Escala de Sintomas Vocais; ISRFL = Índice de Sintomas do Refluxo Faringo-Laríngeo

Foram analisadas as estatísticas descritivas dos valores totais do ESV e do ISRFL nos tipos predominantes de qualidade vocal e, para testar se houve diferença nos valores, foi utilizado um comparativo não paramétrico para cada par. Observaram-se diferenças estatisticamente significativas quando analisados os pares: rugosa-adaptada (p=0,0021) e tensa-adaptada (p=0,0075) no ESV, e rugosa-adaptada (p=0,001) no ISRFL (Tabela 3); constatando relação direta entre os escores aumentados no ESV e ISRFL com o tipo de qualidade vocal.

**Tabela 3:** Comparativo não paramétrico para cada par de parâmetro de qualidade vocal nos valores da ESV e ISRFL

	**Adaptada**	**Rugosa**	**Soprosa**
**ESV**			
**Adaptada**	-		
**Rugosa**	0,0021*	-	
**Soprosa**	0,0799	0,7999	-
**Tensa**	0,0075*	0,2588	0,5192
**ISRFL**			
**Adaptada**	-		
**Rugosa**	<0,001*	-	
**Soprosa**	0,4440	0,5197	-
**Tensa**	0,0948	0,8407	0,1039

Método de Wilcoxon

* Diferenças estatisticamente significativas, com alfa em 0,05

**Legenda:** ESV = Escala de Sintomas Vocais; ISRFL = Índice de Sintomas do Refluxo Faringo-Laríngeo

## DISCUSSÃO

O presente estudo verificou o desvio da qualidade vocal, sintomas vocais e índice de sintomas de refluxo em pacientes com diagnóstico clínico sugestivo de RLF atendidos em uma clínica de otorrinolaringologia na cidade de Lajeado – RS e, a partir dos resultados obtidos, analisou estatisticamente os dados e as possíveis correlações entre eles.

Muitos estudos foram realizados desde a primeira publicação sobre o RLF, mas as controvérsias a respeito da patologia permanecem. Estudo recente considera incompleta a definição até então relatada do RLF, pois acredita que a alteração causada não contempla apenas a mucosa laringofaríngea, mas sim toda mucosa do trato aerodigestivo. Como origem multifatorial dos sintomas, também considera que seja o resultado da sinalização neuroreflexiva e respostas vagais compensatórias^(21)^.

A relação entre refluxo e voz está em evolução nos últimos 40 anos e é cada vez mais comum atribuir as alterações vocais a esta patologia, principalmente na ausência de outras etiologias óbvias. Até o momento o que se sabe é que existe uma relação entre eles^(22)^. Estudo^(23)^ realizado com 121 indivíduos apontou registro de grau geral alterado, soprosidade, tensão e instabilidade no grupo com presença de sintomas de RLF. Além desses achados, o presente estudo também encontrou um maior número de pacientes com vozes desviadas, com voz predominantemente rugosa e grau de alteração leve.

Nesta pesquisa, houve maior ocorrência de pacientes do sexo feminino, tal como relata a literatura que observou que mulheres apresentam maior queixa de refluxo que os homens^(24)^. A média de idade das mulheres com vozes desviadas foi de 41,1 anos e dos homens 47,2, concordando com o estudo que verificou que indivíduos mais velhos têm uma probabilidade maior de apresentarem achados de RLF comparados a sujeitos mais jovens^(25)^.

O RLF afeta o comportamento laríngeo e como consequência da irritação química do material refluído, sinais clínicos como edema de mucosa e hiperemia são encontrados em exames laringológicos^(26)^. Concordando com a literatura, observamos que 98% dos pacientes deste estudo apresentaram edema de prega vocal e 95% hiperemia.

Entre as principais queixas referidas pelos pacientes com RLF, a rouquidão é um dos principais sintomas e ela pode estar presente em até 50% dos sujeitos com alterações vocais. Além deste, também relatam pigarro e tosse crônica^(5)^. Em outro estudo com 39 indivíduos com sinais de RLF, mais de 70% referiram sintomas como pigarro e disfonia^(27)^. Analisando de forma individual cada questão dos protocolos aplicados neste estudo (ESV e ISRFL), observou-se que os itens relacionados ao pigarro foram referidos em maior intensidade e frequência pelos pacientes, em ambas as avaliações. O pigarro pode ser causado por edema na região retrocricóidea e gerar aumento da inflamação local, comprometendo a função fonatória e podendo formar úlceras ou granulações devido ao contato na região dos processos vocais^(9)^.

No ESV, a maior média (2,25) é referente à questão 7, *“você tosse ou pigarreia?”,* seguida pela 19 (2,14), *“você tem muita secreção ou pigarro na garganta?”* e da questão 11 (1,75) *“você sente alguma coisa parada na garganta?”.* Das dez perguntas que mais influenciaram para a piora dos sintomas vocais, seis foram da dimensão físico, ou seja, as questões referentes diretamente à voz (dimensão limitação) não intervieram no aumento do escore total do ESV, justificando os achados que alguns pacientes identificados com vozes adaptadas apresentaram resultados alterados neste protocolo e a complexidade ao tratarmos desse assunto. Já no ISRFL^(16)^, a maior média (3,20) é referente ao item *“pigarro”* – questão 2, seguida da questão 3 *“secreção excessiva na garganta ou nariz”* (2,87). Estes resultados vão ao encontro com os achados encontrados em estudo^(28)^onde utilizou-se o mesmo protocolo para avaliação e constatou-se que as mesmas questões corresponderam a 85,7% e 82,9% dos sintomas, respectivamente. O alto número de sujeitos encontrados com escore alterado era esperado, pois como critério de inclusão no estudo utilizou-se a presença de sinais e sintomas sugestivos de RLF.

A associação entre sinais sugestivos de RLF e a presença de alterações vocais ainda é controversa. Assim como nessa pesquisa, os estudos apontam para uma relação, mas não comprovam a relação causal entre eles^(5,29,30)^. A vasta diferença que existe entre os estudos sobre RLF, no que diz respeito à epidemiologia, sinais clínicos, diagnóstico e tratamento, dificulta que seja estabelecida uma única conclusão sobre o assunto. Ao utilizarmos o exame laringológico em vez da pHmetria para identificar sinais sugestivos de refluxo, podemos ter gerado uma limitação em nosso estudo já que esta última é considerada padrão ouro no diagnóstico de refluxo. Porém, o estudo traz uma importante contribuição para a área de relação voz e refluxo laringofaríngeo pelo seu representante número de sujeitos e a forma de avaliação vocal multidimensional, com informações de avaliação laríngea, protocolos de autoavaliação e avaliação perceptivoauditiva da voz, trazendo robustez aos seus resultados.

## CONCLUSÃO

A maior parte dos sujeitos com diagnóstico sugestivo de RLF apresentaram desvios vocais mensurados clinicamente. Indivíduos com vozes desviadas referiram maior ocorrência de sintomas vocais, desvantagem vocal e sensações desagradáveis relacionadas ao RLF mensurados pela ESV e ISRFL.
